# Ovarian Cancer Molecular Stratification and Tumor Heterogeneity: A Necessity and a Challenge

**DOI:** 10.3389/fonc.2015.00229

**Published:** 2015-10-21

**Authors:** Stefan Symeonides, Charlie Gourley

**Affiliations:** ^1^Cancer Research UK Centre, MRC Institute of Genetics and Molecular Medicine, University of Edinburgh, Edinburgh, UK

**Keywords:** ovarian cancer, olaparib, bevacizumab, heterogeneity, stratification

## Introduction

Only two new drugs have been licensed for the treatment of epithelial ovarian cancer in the last 5 years (bevacizumab and olaparib). These are also the only two molecularly targeted agents licensed in this disease. As we continue to move into the genomic era of cancer therapy, it is clear that optimal therapy is going to depend on molecular stratification and that the stratification itself is going to need to contend with tumor heterogeneity. In this article, we discuss molecular stratification and tumor heterogeneity in the context of high-grade serous ovarian cancer.

The development of bevacizumab and olaparib has provided contrasting examples of stratification in molecularly targeted agents. Bevacizumab is licensed as an unselected agent, currently without molecular (or indeed histological) stratification. However, emerging data may be able to help us refine which patients may benefit the most from this agent (and which may not require it). Any such refinement can be expected to increase the median benefit in the selected population and reinforce the cost:benefit advantage. Conversely, olaparib is licensed as a highly selected agent, currently by genomic or somatic *BRCA1/BRCA2* mutation in high-grade serous cancer. However, emerging data may be able to help us expand its role into tumors with other homologous recombination deficits (while also determining if all *BRCA1/BRCA2* mutations respond equally). For both agents, however, cancers progress even on continuous therapy and targeting the resistant clones that have emerged from tumor heterogeneity will be key to extending benefit for these patients.

## Bevacizumab

Bevacizumab is the first vascular endothelial growth factor (VEGF)-targeted therapy to have been licensed in ovarian cancer; by the European Medicines Agency (EMA) in 2011 and the United States Food and Drug Administration (FDA) in 2014. These licenses differ although in both cases the bevacizumab is given in combination with chemotherapy. The EMA license was the result of first line data from the GOG 218 (1) and ICON7 (2) studies, second line platinum sensitive data from OCEANS (3) and second line platinum resistant data from AURELIA (4) all of which demonstrated a statistically significant increase in progression free survival compared to chemotherapy alone. The FDA license relates to the platinum resistant setting only and was dependent on the data from the AURELIA study. The optimal setting for this treatment is unknown (5), as is the value of treating through progression or utilizing combinations of anti-angiogenic therapies. VEGF-targeted therapy is clearly an active approach in ovarian cancer (at least in combination with chemotherapy) and other anti-angiogenic agents have been investigated in this setting including pazopanib (6), cedirinib (7), nintedanib (8), aflibercept (9), trebananib (10), sunitinib (11), sorafenib (12), and (PDGFR) imatinib (13).

Given the mode of action, it was not unreasonable to seek potential broad activity for bevacizumab and, as with all previous agents in ovarian cancer, these trials had no molecularly or histological stratification. However, potential biomarkers are now emerging for benefit from bevacizumab (14), building on the extensive translational work incorporated in ICON7. For blood biomarkers, a link is evident between circulating Ang1 plus Tie2 levels and progression free survival (15), with most of the benefit from bevacizumab in the high Ang1-low Tie2 group (HR 0.27), no significant effect in the low Ang1 group and a detriment in the high Ang1-high Tie2 group (HR 3.6). A possible link noted with plasma VEGF-A in other tumor types (16), was not seen in this ovarian dataset. For tissue biomarkers, a signature made up of tissue mesothelin, FLT4 and AGP and blood CA125 also has potential to strongly differentiate between benefit and harm from bevacizumab but was limited by patient numbers in the analysis (17). Recently, a transcriptomic signature has been presented (18), which identifies distinct molecular subgroups of high-grade serous ovarian cancer that respond very differently to bevacizumab. In this analysis, the two proangiogenic subgroups had a poorer overall survival but appeared to contain all the benefit from bevacizumab. The other, immune subgroup had a superior prognosis but had a detriment (HR 2.0) from bevacizumab. This data will need confirmation in further datasets but the above examples suggest that we are getting closer to a molecular biomarker for bevacizumab benefit (and resistance). The next step will of course be identifying if these molecular signals also emerge in acquired resistance and if they indicate a druggable pathway to improve or extend the activity of VEGF-directed therapies to resistant tumors (or resistant clonal subpopulations). The story may be complicated by the fixed duration of bevacizumab in some studies (such as ICON7) but continuous maintenance therapy is the expected direction of travel for the future.

Hopefully, the above approaches can help address the mystery of why VEGF-directed therapy does not yet seem to be living up to its clear potential. In the phase 2 single agent studies (19, 20), bevacizumab had a roughly 20% objective response rate (ORR), a figure matched by the additional ORR benefit seen compared to chemotherapy alone in phase 2 and phase 3 combination studies, regardless of setting (Table 1). It is unclear why this clear ORR benefit has not translated into more impressive PFS or OS benefits in the phase 3 first line studies (PFS benefit 3.8m in GOG 218 and 2 months in ICON7, with updated analysis of the latter non-significant and no OS benefit in the ITT population). It seems likely that there is a subset benefiting greatly from therapy (and this subset will be evident when response rate is the primary endpoint, as in some phase 2 studies) but that the effect is being diluted by the current lack of a selection biomarker and is therefore harder to detect when PFS is the primary endpoint (as in the phase 3 studies). Identifying this subset may be the key to widening its licensed indications. Identifying the tumor heterogeneity that leads to resistance to VEGF-directed therapy may be the key to improving and prolonging the benefit.

**Table 1:** **Pivotal phase 2 and phase 3 bevacizumab studies in ovarian cancer**.

Study	Patients	% Platinum resistant	Response rate (%)		Median PFS (months)		Median OS (months)	
**SINGLE AGENT PHASE 2 BEVACIZUMAB STUDIES**								
GOG-170D	62	58	21		4.7		17	
Burger et al. (19)
AVF2949	44	100	16		4.4		10.7	
Cannistra et al. (20)^a^

**Study**	**Agents**	**Setting**	**Response rate**		**Median PFS (months)**		**Median OS (months)**	
			**Bev**	**No bev**	**Bev**	**No bev**	**Bev**	**No bev**

**RANDOMIZED PHASE 3 BEVACIZUMAB STUDIES**								
GOG218	Carbo/pac + bev or plac	First line	Unknown		14.1	10.3	39.7	39.3
Burger et al. (1)	(concom and maint)				*P* < 0.001		N.S.	
ICON7	Carbo/pac ± bev maint	First line	67%	48%	19.8	17.4	44.5	44.6
Perren et al. (2)			*P* < 0.001		*P* = 0.04		N.S.	
OCEANS	Carbo/gem + bev or plac	Platinum	78%	57%	12.4	8.4	33.3	35.2
Aghajanian et al. (3)	(concom and maint)	sensitive relapse	*P* < 0.0001		*P* < 0.0001		N.S.	
AURELIA	Chemo vs. chemo plus bev	Platinum resistant relapse	31%	13%	6.7	3.4	16.6	13.3
Pujade-Lauraine et al. (4)			*P* < 0.001		*P* < 0.001		N.S.	

*^a^Study stopped prematurely because five gastrointestinal perforations out of 44 patients*.

*PFS, progression free survival; OS, overall survival; JCO, Journal of Clinical Oncology; bev, bevacizumab; NEJM, New England Journal of Medicine; carbo, carboplatin; pac, paclitaxel; plac, placebo; concom, concomitant; maint, maintenance; gem, gemcitabine; chemo, chemotherapy*.

## Olaparib

Olaparib is the first poly ADP ribose polymerase (PARP)-inhibitor to have been licensed in ovarian cancer (2014). The EMA license is as post-chemotherapy maintenance in patients with germline or somatic *BRCA1* or *BRCA2* mutations. This was as a result of the subgroup analysis (21) of *BRCA1/2* mutant cancers (germline or somatic), in the molecularly unselected Study 19 of relapsed platinum sensitive high-grade serous ovarian cancer (22). The FDA license is as monotherapy in patients with deleterious or suspected deleterious germline BRCA mutated advanced ovarian cancer who have been treated with three or more prior lines of chemotherapy. PARP inhibitors have demonstrated strong activity in molecularly selected populations and other agents include rucaparib (23), niraparib (24), veliparib (25), and talazoparib (26).

The target population was a clear priority from the original phase 1 study, where *BRCA1/BRCA2* mutation carriers were the predominant responders, leading to a *BRCA1/BRCA2* expansion (27), with promising activity in ovarian cancer (28), which was subsequently confirmed in phase 2 (29). However, *BRCA1/BRCA2* mutation is known not to be an absolute biomarker for sensitivity and other homologous recombination defects have been strongly implicated as additional predictive biomarkers (30). Study 19 represented a pragmatic approach to enrich for this whole group without genetic testing by looking at a platinum-sensitive, high-grade serous histopathology, ovarian population (22), given the known link between platinum sensitivity, high-grade serous histopathology and *BRCA1/2* or wider homologous recombination gene defects (31, 32), even in non-hereditary cases (33, 34). However, although this study did identify activity in the non-BRCA mutant subgroup (HR 0.54), the dramatic effect was really in the *BRCA1/2* mutant subgroup (HR 0.18), and the diluted overall signal risked being a barrier to licensing of these important, active agents. Therefore, olaparib is now licensed for *BRCA1/2* mutant cancers and steps are being taken to identify additional predictive biomarkers, including other known homologous recombination defects. However, predictive biomarkers are seldom binary and olaparib provides a good example where molecular heterogeneity between patients and within tumors leads to significant variation in activity and resistance.

When comparing different patients with different homologous recombination deficient tumors, we are beginning to realize that all BRCA molecular deficits are not equal – epigenetic changes are clearly a different biology to genetic (30, 32, 35) but what about different specific mutations or the difference between germline and somatic? Today, it is generally accepted that the different histological subgroups of epithelial ovarian cancer represent different diseases (36). However, as with other cancers, it is also clear that different molecular subgroups (30) of high-grade serous ovarian cancer can have different phenotypes (37) and outcomes (38, 39), with homologous recombination defects a clear example. It is natural to extend this to specific homologous recombination proteins and, even further, to specific mutations/defects of individual proteins.

Molecular heterogeneity within an individual’s cancer may be just as important. While adaptive epigenetic changes have been implicated in platinum resistance (40), a more striking mechanism of resistance may underlie some of the cross-resistance of platinum therapy and PARP inhibition that has lead to the focus on platinum sensitive disease in the clinic (28, 41). This is the phenomenon where inactivating mutations within the *BRCA1/BRCA2* genes revert to functional genes (41–45), clearly demonstrating the strong selection pressures, which drive outgrowth of a resistant subclone that lacks the one main feature that defined, or even induced, the original cancer but which subsequently had become its Achilles heel. In effect, the tumor is doing whatever it can to evade the agents used against it, even if that means re-expressing the gene that it had to lose in order to become a cancer cell in the first place.

Of course, not every individual’s tumor will contain the resistant subclones that drive this response and much can be learned from the super-responders (28, 46) who give an example of what we might hope to achieve if we could overcome that heterogeneity.

## The Future

Clearly, future optimal therapy for high-grade serous ovarian cancer will depend on optimal molecular stratification and this is just as true for bevacizumab and olaparib as it will be for future agents. While this will help rise to the challenge of optimizing therapy for inter-patient molecular heterogeneity, monotherapy may never overcome intra-patient heterogeneity. If we want to improve the durability of responses, that pool of resistant clones may need to be narrowed by using combination therapies. Indeed, recent clinical data for the addition of the VEGFR inhibitor, cedirinib, to olaparib have shown a significant increase in response rate and a near-doubling of progression free survival (47). The majority of this benefit was in the BRCA1/BRCA2 wild-type (or unknown) group, perhaps demonstrating that combinations can overcome monotherapy dependencies but also highlighting that there is still a lot to learn about biomarkers for anti-angiogenic and PARP inhibitor agents in ovarian cancer.

## Conflict of Interest Statement

Charlie Gourley has sat on advisory boards for AstraZeneca, Roche, and Nucana. He has also received lecture fees from AstraZeneca and Roche. Through his institution he has received research funding from AstraZeneca, Aprea, and Novartis. Stefan Symeonides declares that the research was conducted in the absence of any commercial or financial relationships that could be construed as a potential conflict of interest.
